# P84 Evaluating a digital newsletter to support ongoing engagement with Antibiotic Guardians

**DOI:** 10.1093/jacamr/dlag102.090

**Published:** 2026-06-26

**Authors:** Miriam Onwunle, Bee Yean Ng, Will Thompson, Charlotte Holtum, Orlagh Quinn, Diane Ashiru-Oredope

**Affiliations:** AMR & HCAI Division, UK Health Security Agency, London, UK; AMR & HCAI Division, UK Health Security Agency, London, UK; Microbiology Society, London, UK; Microbiology Society, London, UK; AMR & HCAI Division, UK Health Security Agency, London, UK; AMR & HCAI Division, UK Health Security Agency, London, UK

## Abstract

**Background:**

The Antibiotic Guardian (AG) campaign is an online campaign launched by UKHSA in 2014, with the purpose of raising awareness and promoting responsible antibiotic use through an online pledge-based model targeting healthcare professionals (HCPs) and the public. As of 2026, the AG campaign has received over 190 000 pledges from 194 countries. However, the pledge represents a single interaction, and sustained engagement mechanisms are needed to reinforce positive messaging. Digital newsletters provide a scalable and resource-efficient approach for reaching a wide and targeted healthcare audience. In 2024, the AG campaign, in collaboration with Microbiology Society launched a quarterly newsletter to support HCPs and organizations across the UK with relevant resources, guidance and updates to support antimicrobial stewardship activities.

**Objectives:**

To evaluate the reach and engagement of the AG newsletter among HCPs.

**Methods:**

Six editions of the Antibiotic Guardian newsletter were disseminated between October 2024 and February 2026 via the GovDelivery platform. The newsletter targets UK-based HCPs and organizations who have pledged and consented to receive communications from 2018, representing a subset of registered Antibiotic Guardians. Engagement metrics, including subscriber numbers, open rates and click rates, were extracted from a Bulletin Detail Report generated by the GovDelivery platform. Data were analysed descriptively using R version 4.3.1. Subscriber demographics, including HCP group, were extracted from the AG pledge database and analysed descriptively.

**Results:**

Across the six editions, the launch edition (October 2024) recorded the highest number of subscribers (*n*=32 392; UK and international). Subsequently, from the second edition onward, the distribution list was refined to include only UK-based subscribers (*n*=23 282) aligning with the focus of the newsletter content and resources. Subscriber numbers then stabilized, with a slight increase observed in edition six in February 2026 (*n*=26 584). All recipients had the option to unsubscribe. Pharmacists comprised the largest subscriber group (33%) followed by pharmacy assistant (29%), pharmacy technicians (10%), nurses (8.2%) and primary care prescribers (4.1%). This mirrors the online pledge data, where the pharmacy workforce represents the largest proportion of AG pledgers. Open rates were consistently above 30% (31%-38%) across all editions, indicating sustained interest among recipients. The click rates were highest in edition one and two at 12% and 15% respectively then declining to 2% in subsequent editions. The first edition included the highest number of links (*n*=53) and links to core antimicrobial stewardship resources were included across editions. The newsletter also encourages recipients to share their stories and experiences which featured in a later edition demonstrating participation beyond passive consumption.

**Conclusions:**

The AG newsletter has demonstrated sustained reach with open rates of 31-38% consistent with healthcare communication benchmarks, supporting its role as a viable digital tool for reinforcing antimicrobial use messaging. The low click rates maybe due to a smaller number of URLs, familiarity with core resources and key messages sufficiently conveyed within the newsletter without requiring further interaction. Future iterations will explore strategies to further enhance engagement such as clearer calls to action and gathering direct feedback from end users.

